# Active role of amino acid metabolism in early diagnosis and treatment of diabetic kidney disease

**DOI:** 10.3389/fnut.2023.1239838

**Published:** 2023-09-14

**Authors:** Chenming Li, Lidong Gao, Chunxiao Lv, Ziqiang Li, Shanshan Fan, Xinyue Liu, Xinyi Rong, Yuhong Huang, Jia Liu

**Affiliations:** ^1^Tianjin University of Traditional Chinese Medicine, Tianjin, China; ^2^Clinical Pharmacology Department, Second Affiliated Hospital of Tianjin University of Traditional Chinese Medicine, Tianjin, China; ^3^College of Traditional Chinese Medicine, Tianjin University of Traditional Chinese Medicine, Tianjin, China; ^4^Tianjin Key Laboratory of Modern Chinese Medicine Theory of Innovation and Application, Tianjin University of Traditional Chinese Medicine, Tianjin, China

**Keywords:** glutamine, taurine, branched-chain amino acids, diabetic kidney disease, review

## Abstract

Diabetic Kidney Disease (DKD) is one of the significant microvascular consequences of type 2 diabetes mellitus with a complex etiology and protracted course. In the early stages of DKD, the majority of patients experience an insidious onset and few overt clinical symptoms and indicators, but they are prone to develop end-stage renal disease in the later stage, which is life-threatening. The abnormal amino acid metabolism is tightly associated with the development of DKD, which involves several pathological processes such as oxidative stress, inflammatory response, and immune response and is also closely related to autophagy, mitochondrial dysfunction, and iron death. With a focus on taurine, branched-chain amino acids (BCAAs) and glutamine, we explored the biological effects of various amino acid mechanisms linked to DKD, the impact of amino acid metabolism in the early diagnosis of DKD, and the role of amino acid metabolism in treating DKD, to offer fresh objectives and guidelines for later early detection and DKD therapy.

## 1. Introduction

Diabetic Kidney Disease (DKD) is one of the most serious microvascular consequences of type 2 diabetes. It is an essential contributor to end-stage renal disease and chronic renal failure. According to the International Diabetes Federation (IDF), roughly 40% of diabetes people experience renal failure, which is ultimately life-threatening (1). The early diagnosis of DKD in clinical is currently characterized by microalbuminuria, decreased glomerular filtration rate (GFR), increased basement membrane thickness, and accumulation of extracellular matrix (2). The pathological biopsy is regarded as the gold standard for determining the presence of DKD.

Many mechanisms caused DKD. Studies have shown that hyperglycemia increases the glomerular filtration rate, causing glucose to accumulate in the kidney and exacerbating renal cell damage. The excess glucose influx into the polyol pathway, where it is converted by aldose reductase (AR) to sorbitol. AR is the rate-limiting enzyme of the polyol pathway. Glucose enters the cell freely without insulin, causing sorbitol concentration rises while intracellular inositol concentration falls. Eventually, it leads to the development of chronic complications of diabetes by interfering with the osmoregulation of cells (3). The polyol pathway also activates protein kinase C (PKC) leading to glomerular dysfunction. While elevated blood sugar speeds up the hexosamine biosynthesis pathway (HBP), causing kidney damage and the production of advanced glycosylation end products (AGEs) (4, 5). In addition, nitric oxide synthase (NOS) of the nitric oxide system can be expressed in the kidney. They can affect renal cellular value-added and lead to renal alterations in diabetic patients (6, 7). Hyperglycemia stimulates NADPH oxidase (NOX) to produce superoxide (SOD), and endothelial NO synthase (eNOS), while the amount of SOD can increase with the decrease of eNOS expression (8, 9). Through the nitric oxide system, SOD can increase and promote oxidative stress. Another mechanism of DKD production is the inflammatory response. Multiple cytokines in inflammatory diseases, both IL-6 and I-8 can alter glomerular permeability by promoting glomerular cell proliferation, producing varying degrees of renal injury. TGF-1 has been identified as a key player in the progression of renal fibrosis, thus activation of inflammatory cytokines stimulates renal injury in DKD (10). By the way, with the gradual development of molecular and cellular fields, epigenetic modifications, mitochondrial dysfunction, autophagy, and iron death have provided other new directions for the mechanism of action of DKD (11).

In this process, we find that various amino acids play important roles in different ways. Amino acids are essential for life activities and have biological functions such as synthesizing proteins, metabolizing energy substances, and acting as precursors for compounds. Amino acids created through the digestion and absorption of dietary proteins can be deaminated to produce alpha-keto acids and urea and decarboxylated to produce various amines. Non-coding amino acids are not involved in protein synthesis, such as cysteine and taurine, etc. According to the classification of nutrients, coding amino acids can be divided into essential and non-essential amino acids, of which nine are essential for human life: leucine, isoleucine, lysine, phenylalanine, valine (Val), tryptophan (Trp), methionine, leucine, isoleucine, and histidine (His), while the non-essential amino acids are aspartic acid (Asp), glutamic (Glu), alanine, and Serine (Ser) (12). It has been shown that glutamine (Gln) is involved in the HBP pathway and the synthesis of AGEs as an energy substrate in the pathogene essential amino acids sis of DKD, while it can alleviate oxidative stress due to iron death through anti-inflammatory and antioxidant effects (13, 14). eNOS can produce endothelial nitric oxide (NO) and L-citrulline (Cit) with the participation of L-arginine. Arginine and Cit are also involved in the hemodynamic nitric oxide system with taurine. While taurine is also a key factor leading to mitochondrial dysfunction. Insulin resistance and the metabolism of branched-chain amino acids(BCAAs) are intimately connected, where leucine (Leu) is also correlated with autophagy in foot cells (15, 16).

With the continuous development of histology and molecular technology, DKD has been studied in more depth in terms of pathogenesis, early diagnosis, and treatment. Additionally, there is growing proof that amino acid metabolism plays a significant role in numerous areas of the early diagnosis and treatment of DKD, especially with the advent of histological and molecular tools (17–19). However, the role of amino acids in the latest research on DKD in terms of pathogenesis, diagnosis, and treatment is still lacking in systematic sorting and introduction. This paper focuses on the non-coding amino acid taurine, the essential amino acid BCAAs, and the nonessential amino acid glutamine acid from the perspective of different kinds of amino acids, and discusses the relationship between different amino acid metabolism and DKD from the perspectives of the mechanism of action, early diagnosis, and treatment, to offer fresh perspectives and approaches for DKD diagnosis and therapy.

## 2. Biological effects of amino acids

### 2.1. Biological effects of taurine

A non-essential amino acid called taurine (NH2CH2CH2SO3H) is only produced in the liver from methionine or cysteine (20). Currently, taurine is regarded as a cytoprotective molecule that has a major role in maintaining glutathione stores, upregulating antioxidant responses, increasing membrane stability, and eliminating inflammatory responses to maintain homeostasis *in vivo* (21). Taurine is a substrate for mitochondrial tRNA and has antioxidant properties. A more recent study by Fakruddin et al. demonstrated that mitochondrial tRNA modifications lacking taurine modifications lead to mitochondrial dysfunction and activate cellular oxidative stress (22). Also, taurine may inhibit oxidative stress by reducing calcium overload, increasing Antioxidant glutathione (GSH) levels, stabilizing biofilms, and inhibiting reactive oxygen species (ROS) accumulation by regulating antioxidant enzymes (21, 23, 24). Second, taurine might be crucial in the inflammation brought on by oxidative stress. The neutrophil myeloperoxidase (MPO)-halogenated mechanism produces hypochlorous acid (HOCL) at the site of inflammation, which taurine reacts with and detoxifies. As a result, less toxic taurine chloramine (TauCl), a more stable and less toxic anti-inflammatory mediator, is created (25). Taurine also has an anti-apoptotic action, which has been shown to counteract apoptosis in several ways, including inhibition of calcium overload and protection of mitochondrial membranes (15, 26).

### 2.2. Biological effects of branched chain amino acids

The three essential amino acids leucine (Leu), isoleucine (Ile), and valine (Val) make up branched BCAAs. In mammals, branched-chain aminotransferases (BCATs) first transaminate all three BCAAs to produce Gln and branched-chain α-keto acids (BCKAs). Then, the branched-chain -keto acid dehydrogenase complex (BCKDH), which is found on the inner surface of the inner mitochondrial membrane and catalyzes the oxidative decarboxylation, oxidizes the BCAA product, releases CO2, and covalently adds a coenzyme a (CoA) moiety. CoA is a hydrophilic cofactor that combines subsequent tricarboxylic acid (TCA) cycle intermediates from isoleucine and Val acetyl coenzyme A and/or succinyl coenzyme A are trapped in the mitochondria. The BCAT and BCKDH processes are shared by all three BCAAs (27, 28). The cellular processes of protein synthesis and epigenetic control can both be impacted by the metabolism of BCAAs. First, Leu has the ability to control mTORC1, a protein kinase that plays a crucial role in controlling cell growth. Leu activates the mTOR pathway, which in turn promotes protein synthesis (29). Energy-demanding tissues (like skeletal muscle) typically see an increase in glucose uptake when leu- and BCAA-induced protein synthesis occurs, which may help to improve insulin signaling, and glucose uptake (30).

### 2.3. Biological effects of glutamine

Glutamine (Gln) is the most prevalent and versatile amino acid in the body, with plasma and tissue Gln concentrations 10 to 100 times higher than those of other amino acids (31). Gln plays a crucial role as an energy substrate in cell proliferation, antioxidant, and anti-inflammatory (32). GSH can directly respond to ROS. Gln, as one of the precursors of GSH, affects the redox status of cells through the glutamine-glutathione pathway. Cruzat et al. showed that an increased glutamine/glutamate ratio enhances the substrate availability for GSH synthesis (33). Gln can also maintain cellular homeostasis *in vivo* and promote cell survival by enhancing intracellular heat shock protein (HSP) levels (34). HSPs are a series of peptide proteins, a particular collection of genes that produce cytoprotective proteins. The organisms generate heat shock (HS) responses by altering HSP gene expression, which response to adverse conditions through stress-related heat shock genes, such as heat shock, oxidants, inflammation, etc. (35).

Another biological effect of Gln is its involvement in the synthesis of the hexosamine pathway. One of the mechanisms for the metabolism of glucose is the HBP, in which glutamine-fructose-6-phosphatase aminotransferase (GFAT) catalyzes the glycolytic pathway’s first conversion of glucose to fructose-6-phosphate (36). Fructose-6-phosphate is mixed with Gln to produce glucosamine-6-phosphate (GlcN-6-P), which is then transformed to N-acetylglucosamine-6-phosphate (GlcNAc-P) by glucosamine-phosphate N -acetyltransferase (GNPNAT) to N-acetylglucosamine-6-phosphate (GlcNAc-6-P). Then to N-acetylglucosamine-1-phosphate (GlcNAc-1-P) via phosphoglucose metastases 3 (PGM3), followed by the enzymatic synthesis of uridine diphosphate N-acetylglucosamine (UDP-GlcNAc). Finally, the binding of O-GlcNAc to protein Ser is promoted or hydrolyzed by O-GlcNAc transferase (OGT) and O-GlcNAcase (OGA), which in this way leads to the deposition of various types of collagen and affects the function of the protein (13). Studies have revealed that the development of DKD, insulin resistance, and decreased insulin secretion are all directly related to increased metabolism of this route (37).

## 3. The role of amino acid metabolism in the early diagnosis of DKD

A study technique called metabolomics is used to analyze tiny molecules created by biological systems made up of cells, tissues, fluids, and organisms in both qualitative and quantitative ways (38). For the purpose of looking for illness diagnostic indicators, liquid-phase mass spectrometry is used to systematically detect the alterations in small molecule metabolite patterns (39). At present, patients with DKD are mostly in the urine protein-positive phase upon diagnosis, and irreversible damage to the renal tubules has already occurred. Consequently, the prognosis is dismal and patients’ quality of life is severely compromised (40). DKD patients have a variety of abnormalities in their digestion of sugar, lipids, and amino acids. Metabolomics techniques can be used to identify early diagnostic markers of the disease by observing changes in the small molecule products of metabolic pathways in both the urine and serum of DKD patients (19). The actions are summarized below and shown in Table 1.

**Table 1:** Metabolomic study of various amino acids in early diagnostic markers of DKD.

Amino acid	Study	Conditions	Sample	Main results
Trp	(41)	DKD patients (*n* = 158)	Serum	Increased C-glycosyltryptophan was associated with a faster glomerular filtration rate (eGFR) slope and also with decreased renal function and risk of end-stage renal disease.
	(42)	T2DM patients (*n* = 286)	Serum, urine	The serum metabolite C-glycosyltryptophan predicted a decrease in eGFR at follow-up and improved the predictability of clinical parameters.
	(43)	PKD patients (*n* = 95)	Serum, urine	Metabolites such as threonine, Trp, methionine, homovanillic acid sulfate and Cit can be used as potential biomarkers for early diagnosis.
Gln	(44)	CKD patients (*n* = 15)	Urine	Significant increases in glutamate, guanyl acetate, α-phenylacetylglutamine and N-oxotrimethylamine.
	(19)	T2DM patients (*n* = 90)	Serum, urine	A significant decrease in serum Gln levels indicates that Gln may be used as one of the early diagnostic markers for DKD.
	(45)	DKD patients (*n* = 70)	Urine	The γ-GGT appeared significantly elevated in the massive albuminuria group, and the γ-GGT could be abnormal earlier than in microproteinuria.
BCAAs	(46)	Health volunteers (*n* = 1,680)	Serum	BCAAs are closely related to insulin resistance, and plasma levels of BCAAs can be used as one of the early diagnostic markers of diabetes.
	(19)	T2DM patients (*n* = 90)	Serum, urine	Amino acid levels tended to increase with disease progression, with BCAAs being the most prominent.
	(47)	Healthy, T2MD, DKD patients	Serum, urine	High levels of Val and isoleucine may be a risk factor for the development of DKD.
	(48)	T2DM patients (*n* = 201)	Serum	BCAA and phenylalanine are highly associated with diabetes, demonstrating the potential importance of amino acids in the early stages of diabetes development.
	(49)	T1MD patients (*n* = 637)	Serum	Isoleucine, leucine and Val had significant positive cross-sectional correlations with eGFR, indicating a strong relationship between BCAAs and diabetes.
Cit and Orn	(50)	ESRD patients (*n* = 55)	Serum	Concentrations in the urea cycle such as arginine, Asp., Cit and Orn were significantly increased.
	(40)	T2DM Patients (*n* = 127)	Serum	Cit is independently associated with the occurrence of diabetes mellitus combined with microproteinuria, and therefore Cit can be used as one of the early diagnostic markers of DKD
	(45)	PKD patients (*n* = 95)	Serum, urine	The sensitivity of Cit in blood and urine is the highest in early diagnosis.

### 3.1. The essential amino acids

#### 3.1.1. Branched chain amino acids

Increased levels of BCAA in the blood have been linked to the emergence of insulin resistance, according to studies. BCAAs may encourage better metabolic phenotypes, such as increased insulin sensitivity and glucose uptake (51). Insulin resistance and BCAAs have a mutually reinforcing interaction. The impaired function of BCAT and BCKDH, important pathways in the metabolism of insulin resistance, is thought to be a primary genetic abnormality. The accumulation of related metabolites and branched-chain ketone acids after elevated insulin levels may lead to further insulin resistance (52). High levels of BCAA polymorphisms are linked to a high risk of type 2 diabetes, according to a Mendelian randomization study that used genes related to BCAA metabolism to evaluate the causality of an influence on insulin resistance (53). Another possible mechanism is related to the activation of mTOR. BCAA overload may activate the mTOR pathway and inhibit the PI3K-Akt intracellular pathway, causing insulin resistance (29, 54). mTOR is also a key factor in inhibiting autophagy in renal tubular epithelial cells, and Leu may regulate cellular autophagy through the mTOR pathway to mitigate renal injury (55). Additionally, BCAA can sustain cellular energy metabolism, control mitochondrial function, and lower ROS generation to decrease oxidative stress through BCAT, which in turn lessens DKD.

According to Garibotto et al., renal failure can lower the glomerular filtration rate, which in turn can cause acidosis and impact the catabolism of BCAAs. As DKD continues, it can also cause a decline in BCAA levels because of kidney damage (56). Therefore, changes in branched-chain amino acid levels can be consistent with the progression of DKD and also demonstrated that BCAAs can be used as one of the early diagnostic markers of DKD. Wang et al. showed that plasma levels of BCAAs can be used as one of the early diagnostic markers of diabetes by metabolomics liquid chromatography–tandem mass spectrometry (48). Also, BCAAs are closely related to insulin resistance, which can lead to the development of DKD, and therefore elevated levels of BCAAs can be detected in patients with DKD (40). In a prospective study of type 2 diabetic patients, Zhu et al. hypothesized that high levels of Val and isoleucine may be one of the risk factors for the development of DKD (47). Wang et al. study concluded by metabolomic analysis that serum branched-chain amino acid levels decreased with disease progression, suggesting that elevated BCAA could predict the development of diabetes (19). By using metabolomic analysis on 637 individuals with type 1 diabetes, Tofte et al. established a strong positive cross-sectional connection between the BCAAs isoleucine, leucine, Val, and eGFR. They came to the conclusion that BCAAs are intimately associated with diabetes (49).

#### 3.1.2. Tryptophan

Tryptophan (Trp), one of the aromatic amino acids, is crucial for the early detection of DKD. Niewczas et al., found that greater levels of C-glycosyl tryptophan, O-sulfotyrosine, and N-acetyl threonine were linked to quicker glomerular filtration rate (eGFR) slopes in 158 diabetic patients with proteinuria and chronic renal disease (41). According to Solini et al.’s screening of metabolomics in serum and urine samples from 286 T2DM patients, the combination of serum metabolites (C-glycosyl tryptophan, pseudouridine, and N-acetyl threonine) predicted lower eGFR and increased the predictability of clinical parameters (42). Zhang et al. performed a metabolomic analysis of 95 patients with urinary albumin nephropathy (PKD). They concluded that metabolites such as threonine, Trp, methionine, homovanillic acid sulfate, and Cit could be potential biomarkers for early diagnosis (43).

### 3.2. The non-essential amino acids

#### 3.2.1. Glutamine

By conducting a meta-analysis of metabolites, Roointan et al. discovered that Gln in the blood is one of the most significant non-invasive early diagnostic biomarkers and that several important metabolites, including glutamate, arginine, glycine, and Gln, are highly centralized (57). According to urine metabolomics research performed by Feng et al., patients with chronic kidney disease had a large rise in the metabolite -phenylacetylglutamine (44). Wang et al. demonstrated a significant decrease in serum Gln levels in the urinary albumin group. As a marker of kidney injury, it can indicate that changes in Gln levels can reflect kidney injury in DKD patients and may be used as one of the early diagnostic markers of DKD (44). Zhang et al. showed that serum γ-glutamine transferase (γ-GGT) was significantly elevated in patients in the massive albuminuria group. While γ-GGT could show abnormalities earlier in the absence of trace urine protein. It suggests the significance of Gln in the early diagnosis (45).

#### 3.2.2. Citrulline and ornithine

Following metabolite functional enrichment analysis in DKD, Roointan et al. found that the urea cycle was one of the most variable sets of metabolites (57). The urea cycle also called the ornithine (Orn) cycle, plays a key role in proteolytic metabolism, while insulin can also reduce the biosynthetic capacity of urea (58). Citrulline (Cit) is typically absorbed by the kidneys and converted to urea via arginine. Orn and Cit are all components of the urea cycle. Urea is the major product of nitrogen metabolism and is formed from free ammonia and aspartate (40). Arginine can be transformed into Orn or Cit in the urea cycle, which aids arginase in the production of urea or NO molecules (57). Chuang et al. showed that the concentration of amino acids involved in the urea cycle was significantly increased in patients with end-stage renal disease (50). Jiang et al. came to the conclusion that Cit was independently linked with the development of diabetes mellitus in conjunction with microproteinuria (40). Therefore, Cit can be used as one of the early diagnostic markers of DKD. By analyzing 95 individuals with PKD’s serum and urine using metabolomic techniques, Zhang et al. came to the conclusion that Cit in blood and urine had the highest sensitivity in early diagnosis (43).

## 4. The role of amino acid metabolism in the treatment of DKD

### 4.1. Role of various amino acids in the treatment of DKD

#### 4.1.1. Taurine

Elevated blood glucose leads to a decrease in mitochondrial membrane potentials (MMPs), GSH, and NO levels, prompting the downregulation of eNOX to activate SOD and finally induce DKD through structural damage. Taurine can regulate oxidative stress induced by hyperglycemia to its normal level through antioxidant activity (15). By preventing calcium excess and enhancing mitochondrial respiratory performance, taurine was demonstrated by Zhang et al., to minimize the formation of ROS in the mitochondria (26).

**Figure 1:**
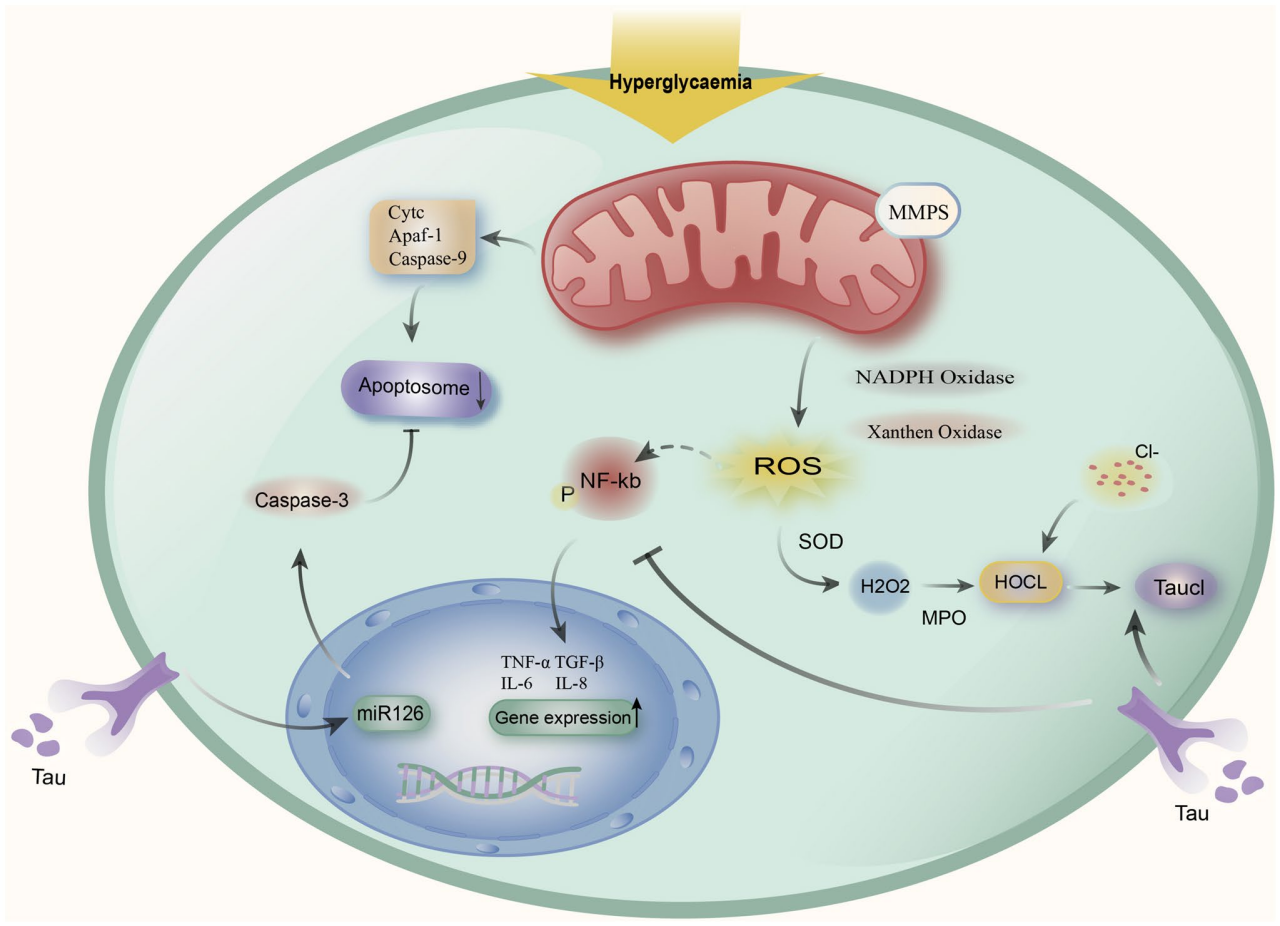
Taurine alleviates the mechanism of action of DKD. Hyperglycemia causes mitochondria to release cytochromes (Cytc), apoptotic protease activating factor 1 (Apaf-1), and caspase-9 to produce apoptotic complexes to induce apoptosis, and taurine regulates the miR-126 gene to alleviate apoptosis. At the same time, hyperglycemia induces the decline of mitochondrial Membrane potentials (MMPs) and activates Superoxide SOD to stimulate oxidative stress, oxidative stress activates NF-kb expression, upregulates IL-6, IL-8, TGF-β1, and other pro-inflammatory factors, taurine can reduce the release of inflammatory factors by inhibiting NF-kb. Meanwhile, by halogenating hypochlorous acid (HOCL) created by the neutrophil myeloperoxidase (MPO) reaction with taurine to create taurine chloramine (TauCl), taurine can reduce the inflammatory response.

In addition, it has been demonstrated that oxidative stress can activate NF-kb, which can increase the expression of pro-inflammatory factors like IL-6, IL-8, and TGF-1. Taurine inhibits the expression of pro-inflammatory factors by reducing ROS and NF-kb activity, which is then involved in reducing their inflammatory response brought on by oxidative stress (15). TauCl has bactericidal and anti-inflammatory properties. It can inhibit pro-inflammatory cytokine production, which in turn is involved in alleviating its inflammatory response induced by oxidative stress (25). Meanwhile, DKD alters apoptotic markers, and hyperglycemia induces the mitochondria to release cytochrome C (Cytc) and produce apoptotic complexes, which in turn promotes apoptosis. Taurine can regulate the apoptotic parameters of apoptotic markers through the protective and anti-apoptotic effects of mitochondrial membranes. Thus reducing the structural damage of renal cortex caused by DKD, and Xie et al. showed that taurine can alleviate endothelial cell apoptosis by regulating miR-126 gene expression (Figure 1) (15, 59).

Secondly, taurine supplementation can prevent insulin resistance due to hyperglycemia through its antioxidant effect, anti-inflammatory response, and reduction of apoptosis. It can also treat DKD by reducing the production of proteinuria and inhibiting glomerular hypertrophy and tubulointerstitial fibrosis (60). Taurine, cysteine, and methionine are amino acids that contain sulfur and can be used as adjuvant therapy to prevent diabetes and its associated problems. According to research by Manna et al., eNOX produced by the nitric oxide system can induce oxidative stress, which can be alleviated by taurine through antioxidant effects (61). While arginine is a precursor of NO and the nitric oxide pathway inhibition of the nitric oxide pathway can lead to systemic hypertension, reduced glomerular filtration rate and effective renal flow. Arginine supplementation can alleviate glomerulosclerosis and thus delay renal injury (62).

#### 4.1.2. Glutamine

First, the antioxidant and anti-inflammatory properties of Gln may be responsible for its nephroprotective effects in DKD. Nasri et al. have shown that oral Gln can reduce renal inflammation as well as oxidative stress biomarker levels in type 2 - diabetic rats, while improving pathological changes such as glomerulosclerosis and leukocyte infiltration (63). Meanwhile, in DKD, the production of large amounts of SOD induces iron death in the renal cell membrane through lipid peroxidation. GSH can play an antioxidant role through the GPX4 enzyme to reduce oxidative stress caused by iron death (11).

**Figure 2:**
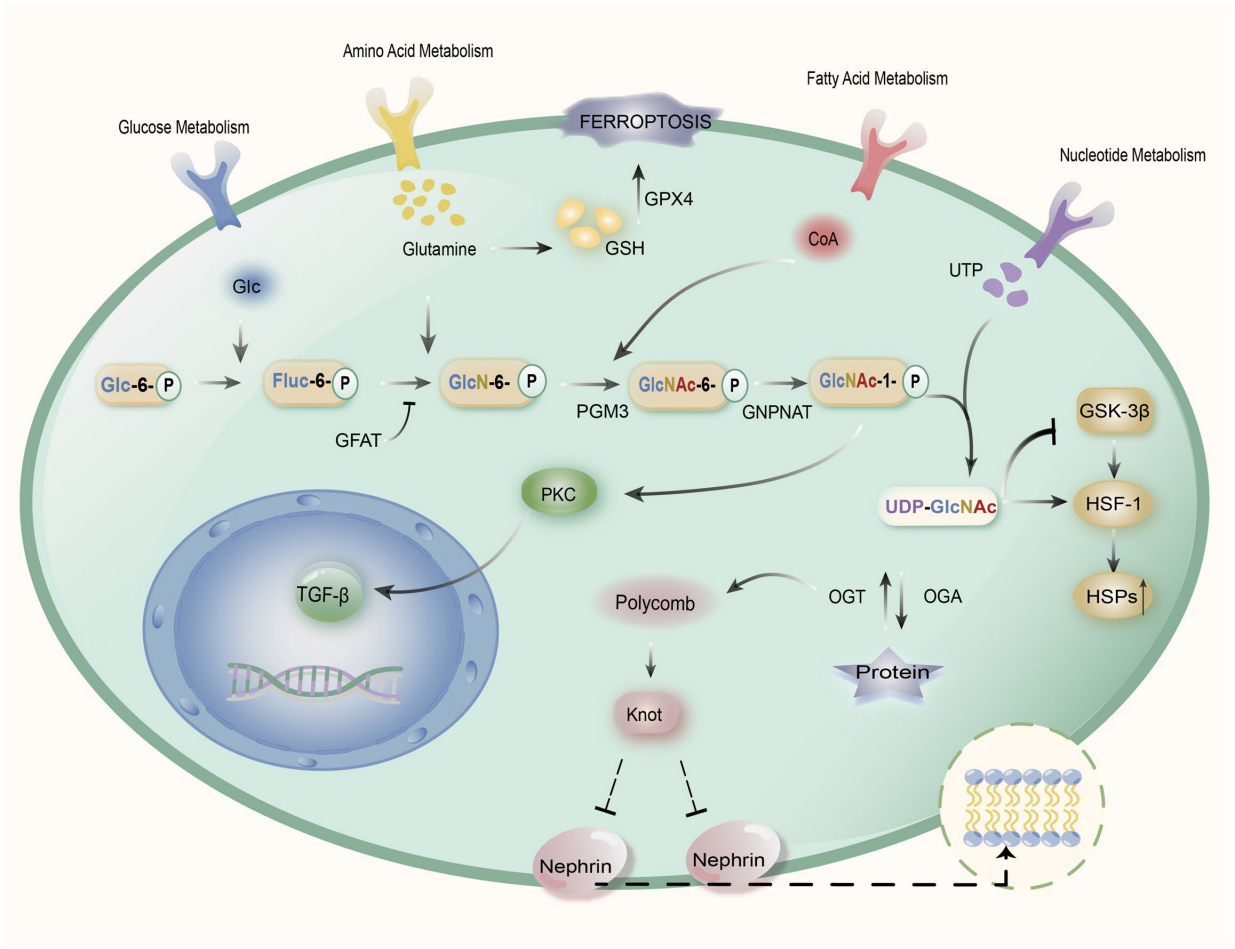
Mechanism of action of Gln in DKD. Gln induces the GPX4 enzyme through the synthesis of glutathione (GSH) to alleviate oxidative stress caused by iron death. Meanwhile, glucose generates fructose-6-phosphate (Fruc-6-P) through glycolysis, which later synthesizes glucosamine-6-phosphate (GlcN-6-P) with Gln, and GlcN-6-P is transformed to N-acetylglucosamine-6-phosphate (GlcNAc-6-P) catalyzed by glucosamine phosphate N-acetyltransferase (GNPNAT) through phosphate glucose metastases 3 (PGM3) to N-acetylglucosamine-1-phosphate (GlcNAc-1-P), followed by an enzymatic reaction to generate uridine diphosphate N-acetylglucosamine (UDP-GlcNAc), which can mediate renal cell dysfunction via the OGT-Polycomb-Knot-Sns pathway, while inhibiting synthesis of glycogen synthase GSK-3β, which can release HSF1 receptors, thereby enhancing heat shock gene expression and stimulating inflammatory responses. In addition, GlcNAc-1-P can activate protein kinase C (PKC), which in turn stimulates TGF-β gene expression and damages renal cells. The hexosamine pathway’s rate-limiting enzyme is GFAT, and through the regulation of Glutamine-fructose-6-phosphatase aminotransferase (GFAT), it can reduce TGF-β expression, as well as reduce the production of UDP-GlcNAc, which in turn reduces the heat shock inflammatory response and mitigates the extent of renal cell injury.

In addition, the heat shock response through HBP interacts with Gln metabolism (34). Transforming growth factor-β (TGF-β)-upregulation in rat renal mesangial cells has been demonstrated to be correlated with the hexosamine pathway, which is a key factor in the pathogenesis of DKD. It suggests that high glucose levels induce the production of fibronectin, especially in the glomeruli of DKD patients, this effect is eliminated by Gln inhibition through GFAT (64). GFAT is an important rate-limiting enzyme for Gln entry into the hexosamine pathway, and by inhibiting fructose-6-phosphate cells, it can eliminate the effector component of the extracellular matrix of the renal mesangial (37, 64). Glucose metabolism to fructose-6-phosphate enters into the hexosamine pathway, which is under the control of GFAT to produce the ultimate product UDP-GlcNAc. UDP-GlcNAc is a high-energy complex that regulates energy homeostasis and promotes insulin resistance in glucose transport, insulin secretion, and gene expression (36, 65). In renal cells, UDP-GlcNAc mediates renal cell dysfunction through the OGT-Polycomb-Knot-Sns pathway (66). Meanwhile, Leite et al. showed that UDP-GlcNAc can also inhibit glycogen synthesis by blocking the glycogen synthase GSK-3β. On the other hand, it can release HSF1 receptors, thus enhancing the heat shock gene expression and stimulating the inflammatory reaction as a way to induce diabetic nephropathy (Figure 2) (34).

Gln, as the most important intermediate in gluconeogenesis and amino acid metabolism, can improve insulin resistance and reduce renal injury through the hexosamine pathway. Additionally, it can promote gluconeogenesis, control glucose metabolism, and prevent protein hydrolysis in the liver and skeletal muscle, promoting protein synthesis in those tissues (67). Perriello et al. showed that the normal absorption of Gln in the population can regulate gluconeogenesis *in vivo* (68). Hu et al. demonstrated that Gln by administration attenuated sepsis-induced renal injury in mice (69). As scavengers of reactive oxygen species (ROS), aspartate and glutamate help lessen the oxidative stress and inflammatory response brought on by DKD (70).

#### 4.1.3. Other amino acids

A dipeptide called myostatin is produced in living cells from β-alanine and L-histidine. It can protect podocytes and thylakoid cells through antioxidant and anti-glycosylation properties, thereby alleviating DKD and slowing down aging (71). Angelica et al. showed that myostatin affects glomerular intracellular pressure through vasomodulation of afferent small arteries, thereby alleviating renal injury in DKD (72). Peters et al. demonstrated that myostatin treatment reduced glomerular ultrastructural damage, albuminuria production, and prevented alterations in renal myostatin metabolism in diabetic mice (73). Zhu et al. showed that myostatin could alleviate foot cell injury by targeting cystatin-1 to inhibit its scorching effect as a way to alleviate DKD (74).

Furthermore, different kinds of amino acids might be useful in treating DKD. Luo et al. showed that Orn metabolites can alleviate renal injury by activating activated mTOR signaling and cytoskeletal remodeling in podocytes, and glycine can reduce proximal tubular injury produced by hypoxia by increasing glomerular filtration rate (75). Yatzidis et al. demonstrated that supplementation with glycine, Asp., Glu, His, and arginine increased glomerular filtration rate in normal subjects or in some patients with renal failure. By the way, cysteine and acetylcysteine regulated insulin secretion and plasma glucose levels. Methionine increased mitochondrial DNA density in skeletal muscle and improved insulin sensitivity (61, 67). Tyrosine, Trp, and phenylalanine are low aromatic amino acids that Barba et al. demonstrated reduced renal damage and prevented renal fibrosis in mice (76). Acetylcysteine has been shown by Li et al. to maintain mitochondrial redox homeostasis by activating the SIRT3-SOD2/Gpx4 pathway, which reduces iron death in DKD (77).

### 4.2. Drug treatment of DKD through various amino acids

Emphasis on nephroprotective medicines in the family of anti-diabetic sodium-glucose cotransport protein 2 (SGLT2) inhibitors has gradually shifted in recent years away from its anti-glycemic properties (78). Gong et al. showed that the SGLT2 inhibitor-empagliflozin could mitigate renal injury in DKD through protective effects related to catabolism and transport of BCAAs (79).

Inhibitors of the prolyl hydroxylase enzyme for the hypoxia-inducible factor (HIF), boost the production of erythropoietin in the body naturally and are potentially effective new treatments for anemia in chronic kidney disease. Hasegawa et al. indicated that the HIF stabilizer Enarodustat (JTZ-951) can improve renal tissue oxidative stress and glutathione to glutathione disulfide ratio in diabetic rats, contributing to renal energy metabolism that takes place in early DKD (80).

Anthocyanins (ANT) are polyphenolic substances present in a variety of foods. Li et al. concluded that anthocyanins could alleviate renal injury in DKD by upregulating taurine, Trp, and tyrosine pathways through histological analysis of kidney and serum in mice (81).

Moreover, many herbs can relieve the complications of diabetes in various ways (82). Rong et al. showed that artemether upregulated the expression of Gln, aspartate, Orn, glycine, His, phenylalanine, and threonine in mice with DKD, which in turn alleviated DKD (83).

Han et al. showed that Yi-Shen-Hua-Shi granule could improve DKD through the intestinal-renal axis, which was enriched to one of the relevant metabolic pathways including phenylalanine, tyrosine, and Trp, etc. (84). Tang et al. showed that Huang-Lian-Jie-Du Decoction had a protective effect on DKD by modulating the AGEs/RAGE/Akt/Nrf2 pathway approach to ameliorate disorders of glucolipid metabolism and renal injury. The potential biomarkers including phenylalanine metabolic processes, Trp in metabolism, metabolism of glucose, etc. (85). The actions are summarized below and shown in Table 2.

**Table 2:** Studies of various amino acids in the treatment of DKD.

Amino acid	Study	Conditions	Sample	Main results	Medicine
Gln	(68)	Health Volunteers	Serum	People who absorb Gln normally can regulate gluconeogenesis in the body.	Gln
	(69)	Mice	Serum, kidney	Administration of Gln attenuates sepsis-induced kidney injury in mice.	Gln
Taurine	(86)	Rat	Serum, urine	Taurine can improve glomerular basement membrane metabolism and renal function to prevent the development of DKD.	Taurine
Taurine, cysteine and methionine	(86)	—	—	Sulfur-containing amino acids such as taurine, cysteine and methionine can be used as complementary therapies to prevent diabetes and related complications.	Taurine, cysteine and methionine
Arginine	(62)	—	—	Arginine supplementation can alleviate glomerulosclerosis and thus delay kidney damage.	Arginine
Myostatin (alanine and His)	(72)	Mice	Serum, kidney	Myostatin affects intra-glomerular pressure through vasoregulation of afferent small arteries and alleviates renal injury in DKD.	Myostatin
	(73)	db/db Mice	Serum, urine	Myostatin treatment reduced glomerular ultrastructural damage and albuminuria production and prevented alterations in renal myostatin metabolism in diabetic mice.	
	(74)	Mice	Urine, kidney	Myostatin can alleviate DKD by targeting cystathione-1 to reduce foot cell damage by inhibiting its scorching effect.	
Orn	(75)	db/db Mice	Urine, kidney	Orn metabolites can mitigate kidney injury by activating activated mTOR signaling and cytoskeletal remodeling in podocytes.	Orn
Glycine	(87)	—	—	High levels of Val and isoleucine may be a risk factor for the development of DKD.	Glycine
Glycine, Asp., Glu acid, His and arginine	(67)	CKD Patients	Serum, urine	Supplementation with glycine, aspartate, glutamate, His and arginine in normal subjects or in some patients with renal failure increases glomerular filtration rate.	Glycine, Asp., Glu acid, His and arginine
Methionine	(61)	—	—	Methionine can also increase mitochondrial DNA density in skeletal muscle and improve insulin sensitivity.	Methionine
Low aromatic amino acids	(76)	Mice	Urine, kidney	Tyrosine,Trp and phenylalanine improve kidney injury as well as prevent kidney fibrosis in mice.	Low aromatic amino acids
Acetylcysteine	(77)	Beagle	Serum, kidney	Acetylcysteine alleviates mitochondrial dysfunction and mitigates iron death in DKD via the SIRT3-SOD2 pathway.	Acetylcysteine
BCAAs	(79)	db/db Mice	Serum, kidney	Engeletin may mitigate renal injury in DKD through protective effects related to catabolism and transport of BCAAs.	Empagliflozin
Glutathione	(80)	Rat	Serum, kidney	Enarodustat improves the ratio of glutathione to glutathione disulfide and alleviates oxidative stress in renal tissues of diabetic animals.	Enarodustat
Taurine, Trp, tyrosine	(81)	Mice	Serum, kidney	Anthocyanins can mitigate renal damage in DKD by upregulating taurine, Trp and tyrosine pathways.	Anthocyanin
Gln, aspartate, Orn, glycine, His, phenylalanine and threonine	(83)	db/db T2DM Mice	Serum, kidney	Artemisinin upregulated the expression of Gln, aspartate, Orn, glycine, His, phenylalanine and threonine in mice, thereby alleviating DKD.	Artemisinin
Phenylalanine,Trp	(85)	—	—	Huang-Lian-Jie-Du Decoction ameliorates disorders of glucolipid metabolism and renal injury by regulating AGEs and other protective effects on DKD.	Huang-Lian-Jie-Du Decoction
Phenylalanine, tyrosine, and Trp	(84)	Rat	Serum, kidney	Kidney-rich granules can improve DKD through the intestinal-renal axis, where one of the relevant metabolic pathways enriched to include phenylalanine, tyrosine and tryptophan.	Yi-Shen-Hua-Shi granule

## 5. Conclusion

Different kinds of amino acids play different roles in the diagnosis and treatment of diabetes nephropathy. DKD is difficult to detect, but the metabolic changes of various amino acids can be used to diagnose DKD earlier, which can buy more time for treatment. The majority of the amino acids used as early diagnostic markers are essential amino acids, with trp and BCAAs receiving the most research. Non-essential amino acids Gln, Cit, and Orn can all be detected by metabolomic methods with various elevated or decreased changes, detecting kidney damage before proteinuria. Meanwhile, non-coding amino acid taurine can exert stronger anti-inflammatory effects through the production of Taucl and taurine supplementation can well alleviate the renal damage of DKD. Empagliflozin and Enarodustat both contain amino acids that are BCAAs and Gln, which can treat DKD.

The essential amino acid BCAAs and the non-essential amino acid Gln are widely involved in the early diagnosis of DKD. At the same time, like the non-coding amino acid taurine, they can be used as separate drugs to treat DKD. However, the role of taurine in early diagnosis has not been explored.

Aside from a few amino acids specifically linked to DKD, a combination of multiple amino acids is the most therapeutically effective. There is a wide variety of amino acids, and more adequate research is needed to explore whether there are other amino acids that have a profound impact on the pathogenesis and early diagnosis of DKD. Moreover, the pertinent materials are now primarily experimental studies, and further clinical research is required to further substantiate and demonstrate the therapeutic benefits of amino acids. It is expected that amino acid metabolism will offer a fresh direction and new ideas for the diagnosis of DKD and establish the groundwork for the discovery of new therapeutic targets with an in-depth investigation of the interaction between various amino acids and DKD.

## Author contributions

All authors listed have made a substantial, direct, and intellectual contribution to the work and approved it for publication.

## Funding

This work is funded by National Major Scientific and Technological Special Project (2015ZX09101043-009), National Natural Science Foundation of China (81903768), Integrative Chinese Medicine Research Project of Tianjin Municipal Commission of Health and Family Planning (2019070) and New Teachers’ Scientific Research project “Young Eagle Program” in Tianjin University of Traditional Chinese Medicine (XJS2022203).

## Conflict of interest

The authors declare that the research was conducted in the absence of any commercial or financial relationships that could be construed as a potential conflict of interest.

## Publisher’s note

All claims expressed in this article are solely those of the authors and do not necessarily represent those of their affiliated organizations, or those of the publisher, the editors and the reviewers. Any product that may be evaluated in this article, or claim that may be made by its manufacturer, is not guaranteed or endorsed by the publisher.

## References

[1] Natesan V Kim S-J. Diabetic nephropathy–a review of risk factors, progression, mechanism, and dietary management. Biomol Ther (Seoul). (2021) 29:365–72. doi: 10.4062/biomolther.2020.204, PMID: 33888647 PMC8255138

[2] Selby NM Taal MW. An updated overview of diabetic nephropathy: diagnosis, prognosis, treatment goals and latest guidelines. Diabetes Obes Metab. (2020) 22:3–15. doi: 10.1111/dom.14007, PMID: 32267079

[3] Garg SS Gupta J. Polyol pathway and redox balance in diabetes. Pharmacol Res. (2022) 182:106326. doi: 10.1016/j.phrs.2022.106326, PMID: 35752357

[4] Dunlop M. Aldose reductase and the role of the polyol pathway in diabetic nephropathy. Kidney Int Suppl. (2000) 58:S3–S12. doi: 10.1046/j.1523-1755.2000.07702.x 10997684

[5] Wu T Ding L Andoh V Zhang J Chen L. The mechanism of hyperglycemia-induced renal cell injury in diabetic nephropathy disease: an update. Life (Basel). (2023) 13:539. doi: 10.3390/life13020539, PMID: 36836895 PMC9967500

[6] Pollock JS Pollock DM. Endothelin, nitric oxide, and reactive oxygen species in diabetic kidney disease. Contrib Nephrol. (2011) 172:149–59. doi: 10.1159/000329054, PMID: 21893996 PMC3957180

[7] Schrijvers BF De Vriese AS Flyvbjerg A. From hyperglycemia to diabetic kidney disease: the role of metabolic, hemodynamic, intracellular factors and growth factors/cytokines. Endocr Rev. (2004) 25:971–1010. doi: 10.1210/er.2003-0018, PMID: 15583025

[8] Meza CA La Favor JD Kim D-H Hickner RC. Endothelial dysfunction: is there a hyperglycemia-induced imbalance of NOX and NOS? Int J Mol Sci. (2019) 20:3775. doi: 10.3390/ijms20153775, PMID: 31382355 PMC6696313

[9] Raman CS Li H Martásek P Král V Masters BS Poulos TL. Crystal structure of constitutive endothelial nitric oxide synthase: a paradigm for pterin function involving a novel metal center. Cells. (1998) 95:939–50. doi: 10.1016/s0092-8674(00)81718-3, PMID: 9875848

[10] Rahimifard M Moini-Nodeh S Niaz K Baeeri M Jamalifar H Abdollahi M. Improvement of the functionality of pancreatic Langerhans islets via reduction of bacterial contamination and apoptosis using phenolic compounds. Iran J Basic Med Sci. (2018) 21:920–7. doi: 10.22038/IJBMS.2018.27718.6753 30524692 PMC6272063

[11] Wu Y Chen Y. Research progress on ferroptosis in diabetic kidney disease. Front Endocrinol (Lausanne). (2022) 13:945976. doi: 10.3389/fendo.2022.945976, PMID: 36246888 PMC9556825

[12] Tabe Y Lorenzi PL Konopleva M. Amino acid metabolism in hematologic malignancies and the era of targeted therapy. Blood. (2019) 134:1014–23. doi: 10.1182/blood.2019001034, PMID: 31416801 PMC6764269

[13] Lam C Low J-Y Tran PT Wang H. The hexosamine biosynthetic pathway and cancer: current knowledge and future therapeutic strategies. Cancer Lett. (2021) 503:11–8. doi: 10.1016/j.canlet.2021.01.010, PMID: 33484754

[14] Rabbani N Thornalley PJ. Advanced glycation end products in the pathogenesis of chronic kidney disease. Kidney Int. (2018) 93:803–13. doi: 10.1016/j.kint.2017.11.034, PMID: 29477239

[15] Ma J Yang Z Jia S Yang R. A systematic review of preclinical studies on the taurine role during diabetic nephropathy: focused on anti-oxidative, anti-inflammation, and anti-apoptotic effects. Toxicol Mech Methods. (2022) 32:420–30. doi: 10.1080/15376516.2021.2021579, PMID: 34933643

[16] White PJ McGarrah RW Herman MA Bain JR Shah SH Newgard CB. Insulin action, type 2 diabetes, and branched-chain amino acids: a two-way street. Mol Metab. (2021) 52:101261. doi: 10.1016/j.molmet.2021.101261, PMID: 34044180 PMC8513145

[17] Han L Zhang J Zhao H. Research advances in the pathogenesis and treatment of diabetic nephropathy. J Mod Med Health. (2021) 37:404–7. doi: 10.3969/j.issn.1009-5519.2021.03.012

[18] Wang Z Lu H Yao Z Wang Q. Research progress on type 2 diabetes mellitus and related complications based on metabonomics. Acad J Shanghai Univ Tradit Chin Medi. (2022) 2022:296–300. doi: 10.16306/j.1008-861x.2022.S1.060

[19] Wang X. *Metabolomic analysis for serum and urine of patients at different stages of diabetic nephropathy*. (2012). Available at: https://oversea.cnki.net/KCMS/detail/detail.aspx?dbcode=CDFD&dbname=CDFD1214&filename=1013124172.nh&uniplatform=OVERSEA&v=IHOTWBWRfPpx6hrkB_sdNw5mEtYqcDAjdd6l4HaqQI-16f2QiSAr37ynZgJifZJs (Accessed September 24, 2022).

[20] Jong CJ Sandal P Schaffer SW. The role of taurine in mitochondria health: more than just an antioxidant. Molecules. (2021) 26:4913. doi: 10.3390/molecules26164913, PMID: 34443494 PMC8400259

[21] Baliou S Adamaki M Ioannou P Pappa A Panayiotidis MI Spandidos DA. Protective role of taurine against oxidative stress. Mol Med Rep. (2021) 24:605. doi: 10.3892/mmr.2021.12242, PMID: 34184084 PMC8240184

[22] Fakruddin M Wei F-Y Suzuki T Asano K Kaieda T Omori A. Defective mitochondrial tRNA taurine modification activates global Proteostress and leads to mitochondrial disease. Cell Rep. (2018) 22:482–96. doi: 10.1016/j.celrep.2017.12.051, PMID: 29320742

[23] Haojun Z Yaoling W Ke Z Jin L Junling W. Effects of NaF on the expression of intracellular ca ^2+^ fluxes and apoptosis and the antagonism of taurine in murine neuron. Toxicol Mech Methods. (2012) 22:305–8. doi: 10.3109/15376516.2012.657259, PMID: 22356551

[24] Jafri AJA Agarwal R Iezhitsa I Agarwal P Ismail NM. Taurine protects against NMDA-induced retinal damage by reducing retinal oxidative stress. Amino Acids. (2019) 51:641–6. doi: 10.1007/s00726-019-02696-4, PMID: 30656415

[25] Marcinkiewicz J Kontny E. Taurine and inflammatory diseases. Amino Acids. (2014) 46:7–20. doi: 10.1007/s00726-012-1361-4, PMID: 22810731 PMC3894431

[26] Zhang R Wang X Gao Q Jiang H Zhang S Lu M. Taurine supplementation reverses diabetes-induced podocytes injury via modulation of the CSE/TRPC6 Axis and improvement of mitochondrial function. Nephron. (2020) 144:84–95. doi: 10.1159/000503832, PMID: 31865328

[27] Neinast M Murashige D Arany Z. Branched chain amino acids. Annu Rev Physiol. (2019) 81:139–64. doi: 10.1146/annurev-physiol-020518-114455, PMID: 30485760 PMC6536377

[28] Sivanand S Vander Heiden MG. Emerging roles for branched chain amino acid metabolism in cancer. Cancer Cell. (2020) 37:147–56. doi: 10.1016/j.ccell.2019.12.011, PMID: 32049045 PMC7082774

[29] Wu S. Liu X. Zhen Y. Sun Jie Zhang Xuefeng Wang Tao (2022). Advance of research on the characteristics and biological functions of branched chain amino acids. J Econ Anim, 2022, 1–6.

[30] Gannon NP Vaughan RA. Leucine-induced anabolic-catabolism: two sides of the same coin. Amino Acids. (2016) 48:321–36. doi: 10.1007/s00726-015-2109-8, PMID: 26476924

[31] Cruzat V Macedo Rogero M Noel Keane K Curi R Newsholme P. Glutamine: metabolism and immune function, supplementation and clinical translation. Nutrients. (2018) 10:1564. doi: 10.3390/nu10111564, PMID: 30360490 PMC6266414

[32] Mills EL Kelly B O’Neill LAJ. Mitochondria are the powerhouses of immunity. Nat Immunol. (2017) 18:488–98. doi: 10.1038/ni.3704, PMID: 28418387

[33] Cruzat VF Bittencourt A Scomazzon SP Leite JSM de Bittencourt PIH Tirapegui J. Oral free and dipeptide forms of glutamine supplementation attenuate oxidative stress and inflammation induced by endotoxemia. Nutrition. (2014) 30:602–11. doi: 10.1016/j.nut.2013.10.019, PMID: 24698353

[34] Leite JSM Cruzat VF Krause M Homem de Bittencourt PI. Physiological regulation of the heat shock response by glutamine: implications for chronic low-grade inflammatory diseases in age-related conditions. Forum Nutr. (2016) 41:17. doi: 10.1186/s41110-016-0021-y

[35] Maity TK Henry MM Tulapurkar ME Shah NG Hasday JD Singh IS. Distinct, gene-specific effect of heat shock on heat shock factor-1 recruitment and gene expression of CXC chemokine genes. Cytokine. (2011) 54:61–7. doi: 10.1016/j.cyto.2010.12.017, PMID: 21266308 PMC3048923

[36] Liu Q Ye F. Hexosamine pathway and energy regulation. Chin J Diabetes. (2010) 18:232–4. doi: 10.3969/j.issn.1006-6187.2010.03.024

[37] Elbein SC Zheng H Jia Y Chu W Cooper JJ Hale T. Molecular screening of the human glutamine–fructose-6-phosphate amidotransferase 1 (GFPT1) gene and association studies with diabetes and diabetic nephropathy. Mol Genet Metab. (2004) 82:321–8. doi: 10.1016/j.ymgme.2004.05.004, PMID: 15308130

[38] Nicholson JK Lindon JC Holmes E. “Metabonomics”: understanding the metabolic responses of living systems to pathophysiological stimuli via multivariate statistical analysis of biological NMR spectroscopic data. Xenobiotica. (1999) 29:1181–9. doi: 10.1080/004982599238047, PMID: 10598751

[39] Huang M Yu S Shao Q Liu H Wang Y Chen H. Comprehensive profiling of Lingzhihuang capsule by liquid chromatography coupled with mass spectrometry-based molecular networking and target prediction. Acupunct Herb Med. (2022) 2:58–67. doi: 10.1097/HM9.0000000000000012

[40] Jiang Z. *Preliminary screening of metabolic markers related to diabetic nephropathy*. (2019). Available at: https://oversea.cnki.net/KCMS/detail/detail.aspx?dbcode=CMFD&dbname=CMFD201902&filename=1019160216.nh&uniplatform=OVERSEA&v=q46DAEmDcMEQUA3xd1Iy_FDSw7EuAusbM7RLAs8ukXvnhkW8DBlwEhX1uynr7_ij (Accessed September 24, 2022).

[41] Niewczas MA Mathew AV Croall S Byun J Major M Sabisetti VS. Circulating modified metabolites and a risk of ESRD in patients with type 1 diabetes and chronic kidney disease. Diabetes Care. (2017) 40:383–90. doi: 10.2337/dc16-0173, PMID: 28087576 PMC5319475

[42] Solini A Manca ML Penno G Pugliese G Cobb JE Ferrannini E. Prediction of declining renal function and albuminuria in patients with type 2 diabetes by metabolomics. J Clin Endocrinol Metabol. (2016) 101:696–704. doi: 10.1210/jc.2015-3345, PMID: 26684276

[43] Zhang W Zhang X Liu T. The role of metabolomics techniques in the diagnosis of proteinuria related kidney disease. Lingnan J Emerg Med. (2022) 27:582–4. doi: 10.3969/j.issn.1671-301X.2022.06.029

[44] Feng Q Shang X Li Z Hu F. Feature analysis of urinary Matabolismin advanced chronic kidney disease. J Mod Lab Med. (2019) 34:93–6. doi: 10.3969/j.issn.1671-7414.2019.01.024

[45] Zhang Y Rao K. Clinical significance of serum γ-glutamine transferase and cystatin C in the diagnosis of diabetic nephropathy. J Guangxi Univ Chin Med. (2020) 23:12–5.

[46] Würtz P Soininen P Kangas AJ Rönnemaa T Lehtimäki T Kähönen M. Branched-chain and aromatic amino acids are predictors of insulin resistance in young adults. Diabetes Care. (2013) 36:648–55. doi: 10.2337/dc12-0895, PMID: 23129134 PMC3579331

[47] Zhu H Bai M Xie X Wang J Weng C Dai H. Impaired amino acid metabolism and its correlation with diabetic kidney disease progression in type 2 diabetes mellitus. Nutrients. (2022) 14:3345. doi: 10.3390/nu14163345, PMID: 36014850 PMC9415588

[48] Wang TJ Larson MG Vasan RS Cheng S Rhee EP McCabe E. Metabolite profiles and the risk of developing diabetes. Nat Med. (2011) 17:448–53. doi: 10.1038/nm.2307, PMID: 21423183 PMC3126616

[49] Tofte N Suvitaival T Trost K Mattila IM Theilade S Winther SA. Metabolomic assessment reveals alteration in polyols and branched chain amino acids associated with present and future renal impairment in a discovery cohort of 637 persons with type 1 diabetes. Front Endocrinol. (2019) 10:818. doi: 10.3389/fendo.2019.00818, PMID: 31824430 PMC6883958

[50] Chuang C-K Lin S-P Chen H-H Chen Y-C Wang T-J Shieh W-H. Plasma free amino acids and their metabolites in Taiwanese patients on hemodialysis and continuous ambulatory peritoneal dialysis. Clin Chim Acta. (2006) 364:209–16. doi: 10.1016/j.cccn.2005.07.001, PMID: 16087168

[51] Gannon NP Schnuck JK Vaughan RA. BCAA metabolism and insulin sensitivity - dysregulated by metabolic status? Mol Nutr Food Res. (2018) 62:1700756. doi: 10.1002/mnfr.201700756, PMID: 29377510

[52] Nagao K Yamakado M. The role of amino acid profiles in diabetes risk assessment. Curr Opin Clin Nutr Metab Care. (2016) 19:328–35. doi: 10.1097/MCO.0000000000000305, PMID: 27380310

[53] Lotta LA Scott RA Sharp SJ Burgess S Luan J Tillin T. Genetic predisposition to an impaired metabolism of the branched-chain amino acids and risk of type 2 diabetes: a Mendelian randomisation analysis. PLoS Med. (2016) 13:e1002179. doi: 10.1371/journal.pmed.1002179, PMID: 27898682 PMC5127513

[54] Bloomgarden Z. Diabetes and branched-chain amino acids: what is the link? J Diabetes. (2018) 10:350–2. doi: 10.1111/1753-0407.12645 29369529

[55] Sakai S Yamamoto T Takabatake Y Takahashi A Namba-Hamano T Minami S. Proximal tubule autophagy differs in type 1 and 2 diabetes. J Am Soc Nephrol. (2019) 30:929–45. doi: 10.1681/ASN.2018100983, PMID: 31040190 PMC6551771

[56] Garibotto G Bonanni A Verzola D. Effect of kidney failure and hemodialysis on protein and amino acid metabolism. Curr Opin Clin Nutr Metab Care. (2012) 15:78–84. doi: 10.1097/MCO.0b013e32834d9df6 22108097

[57] Roointan A Gheisari Y Hudkins KL Gholaminejad A. Non-invasive metabolic biomarkers for early diagnosis of diabetic nephropathy: Meta-analysis of profiling metabolomics studies. Nutr Metab Cardiovasc Dis. (2021) 31:2253–72. doi: 10.1016/j.numecd.2021.04.021 34059383

[58] Wang H Ran J Jiang T. Urea In: Yang B Sands JM, editors. Urea transporters subcellular biochemistry. Dordrecht, Netherlands: Springer (2014). 7–29.

[59] Chen WQ Jin H Nguyen M Carr J Lee YJ Hsu CC. Role of taurine in regulation of intracellular calcium level and neuroprotective function in cultured neurons. J Neurosci Res. (2001) 66:612–9. doi: 10.1002/jnr.10027, PMID: 11746381

[60] Schaffer S Kim HW. Effects and mechanisms of taurine as a therapeutic agent. Biomol Ther. (2018) 26:225–41. doi: 10.4062/biomolther.2017.251, PMID: 29631391 PMC5933890

[61] Manna P Das J Sil PC. Role of sulfur containing amino acids as an adjuvant therapy in the prevention of diabetes and its associated complications. Curr Diabetes Rev. (2013) 9:237–48. doi: 10.2174/1573399811309030005, PMID: 23547683

[62] Reyes AA Karl IE Klahr S. Role of arginine in health and in renal disease. Am J Phys. (1994) 267:F331–46. doi: 10.1152/ajprenal.1994.267.3.F331, PMID: 8092248

[63] Nasri M Adibhesami G Mahdavifard S Babaeenezhad E Ahmadvand H. Exogenous glutamine ameliorates diabetic nephropathy in a rat model of type 2 diabetes mellitus through its antioxidant and anti-inflammatory activities. Arch Physiol Biochem. (2020) 129:363–72. doi: 10.1080/13813455.2020.1828478, PMID: 33021829

[64] Schleicher ED Weigert C. Role of the hexosamine biosynthetic pathway in diabetic nephropathy. Kidney Int. (2000) 58:S13–8. doi: 10.1046/j.1523-1755.2000.07703.x, PMID: 10997685

[65] Zhang H Jia Y Cooper JJ Hale T Zhang Z Elbein SC. Common variants in glutamine:Fructose-6-phosphate Amidotransferase 2 (GFPT2) gene are associated with type 2 diabetes, diabetic nephropathy, and increased GFPT2 mRNA levels. J Clin Endocrinol Metabol. (2004) 89:748–55. doi: 10.1210/jc.2003-031286, PMID: 14764791

[66] Peterson SB Hart GW. New insights: a role for O -GlcNAcylation in diabetic complications. Crit Rev Biochem Mol Biol. (2016) 51:150–61. doi: 10.3109/10409238.2015.1135102 26806492

[67] Yatzidis H. Oral supplement of six selective amino acids arrest progression renal failure in uremic patients. Int Urol Nephrol. (2004) 36:591–8. doi: 10.1007/s11255-004-8782-2, PMID: 15787344

[68] Perriello G Nurjhan N Stumvoll M Bucci A Welle S Dailey G. Regulation of gluconeogenesis by glutamine in normal postabsorptive humans. Am J Phys. (1997) 272:E437–45. doi: 10.1152/ajpendo.1997.272.3.E437, PMID: 9124550

[69] Hu Y-M Pai M-H Yeh C-L Hou Y-C Yeh S-L. Glutamine administration ameliorates sepsis-induced kidney injury by downregulating the high-mobility group box protein-1-mediated pathway in mice. Am J Physiol. (2012) 302:F150–8. doi: 10.1152/ajprenal.00246.2011, PMID: 21921023

[70] Yatzidis H. A new, superior, single and stable, amino acid and bicarbonate-based, glucose-free solution for peritoneal dialysis. Dial Transplant. (2002) 31:143–50.

[71] Jukić I Kolobarić N Stupin A Matić A Kozina N Mihaljević Z. Carnosine, small but mighty—Prospect of use as functional ingredient for functional food formulation. Antioxidants (Basel). (2021) 10:1037. doi: 10.3390/antiox10071037, PMID: 34203479 PMC8300828

[72] Rodriguez-Niño A Pastene DO Hettler SA Qiu J Albrecht T Vajpayee S. Influence of carnosine and carnosinase-1 on diabetes-induced afferent arteriole vasodilation: implications for glomerular hemodynamics. Am J Physiol Renal Physiol. (2022) 323:F69–80. doi: 10.1152/ajprenal.00232.2021, PMID: 35635322

[73] Peters V Schmitt CP Zschocke J Gross M-L Brismar K Forsberg E. Carnosine treatment largely prevents alterations of renal carnosine metabolism in diabetic mice. Amino Acids. (2012) 42:2411–6. doi: 10.1007/s00726-011-1046-4, PMID: 21833769

[74] Zhu W Li Y Zeng H Liu X-Q Sun Y-T Jiang L. Carnosine alleviates podocyte injury in diabetic nephropathy by targeting caspase-1-mediated pyroptosis. Int Immunopharmacol. (2021) 101:108236. doi: 10.1016/j.intimp.2021.108236, PMID: 34653727

[75] Luo Q Liang W Zhang Z Zhu Z Chen Z Hu J. Compromised glycolysis contributes to foot process fusion of podocytes in diabetic kidney disease: role of ornithine catabolism. Metabolism. (2022) 134:155245. doi: 10.1016/j.metabol.2022.155245, PMID: 35780908

[76] Barba C Benoit B Bres E Chanon S Vieille-Marchiset A Pinteur C. A low aromatic amino-acid diet improves renal function and prevent kidney fibrosis in mice with chronic kidney disease. Sci Rep. (2021) 11:19184. doi: 10.1038/s41598-021-98718-x, PMID: 34584168 PMC8479128

[77] Li Q Liao J Chen W Zhang K Li H Ma F. NAC alleviative ferroptosis in diabetic nephropathy via maintaining mitochondrial redox homeostasis through activating SIRT3-SOD2/Gpx4 pathway. Free Radic Biol Med. (2022) 187:158–70. doi: 10.1016/j.freeradbiomed.2022.05.024, PMID: 35660452

[78] Scheen AJ. Sodium-glucose cotransporter type 2 inhibitors for the treatment of type 2 diabetes mellitus. Nat Rev Endocrinol. (2020) 16:556–77. doi: 10.1038/s41574-020-0392-2 32855502

[79] Gong Q Zhang R Wei F Fang J Zhang J Sun J. SGLT2 inhibitor-empagliflozin treatment ameliorates diabetic retinopathy manifestations and exerts protective effects associated with augmenting branched chain amino acids catabolism and transportation in db/db mice. Biomed Pharmacother. (2022) 152:113222. doi: 10.1016/j.biopha.2022.113222 35671581

[80] Hasegawa S Tanaka T Saito T Fukui K Wakashima T Susaki EA. The oral hypoxia-inducible factor prolyl hydroxylase inhibitor enarodustat counteracts alterations in renal energy metabolism in the early stages of diabetic kidney disease. Kidney Int. (2020) 97:934–50. doi: 10.1016/j.kint.2019.12.007, PMID: 32171449

[81] Li Y-X Lu Y-P Tang D Hu B Zhang Z-Y Wu H-W. Anthocyanin improves kidney function in diabetic kidney disease by regulating amino acid metabolism. J Transl Med. (2022) 20:510. doi: 10.1186/s12967-022-03717-9, PMID: 36335368 PMC9636632

[82] Zuo X Yao R Zhao L Zhang Y Lu B Pang Z. Campanumoea javanica Bl. Activates the PI3K/ AKT/mTOR signaling pathway and reduces sarcopenia in a T2DM rat model. Acupunct Herb Med. (2022) 2:99–108. doi: 10.1097/HM9.0000000000000027

[83] Rong G Weng W Huang J Chen Y Yu X Yuan R. Artemether alleviates diabetic kidney disease by modulating amino acid metabolism. Biomed Res Int. (2022) 2022:7339611–8. doi: 10.1155/2022/7339611, PMID: 35601149 PMC9117059

[84] Han C Shen Z Cui T Ai S-S Gao R-R Liu Y. Yi-Shen-Hua-Shi granule ameliorates diabetic kidney disease by the “gut-kidney axis.”. J Ethnopharmacol. (2023) 307:116257. doi: 10.1016/j.jep.2023.116257 36787845

[85] Tang D He W-J Zhang Z-T Shi J-J Wang X Gu W-T. Protective effects of Huang-Lian-Jie-Du decoction on diabetic nephropathy through regulating AGEs/RAGE/Akt/Nrf2 pathway and metabolic profiling in db/db mice. Phytomedicine. (2022) 95:153777. doi: 10.1016/j.phymed.2021.153777, PMID: 34815154

[86] Lin S Yang J Wu G Liu M Luan X Lv Q. Preventive effect of taurine on experimental type II diabetic nephropathy. J Biomed Sci. (2010) 17:S46. doi: 10.1186/1423-0127-17-S1-S46, PMID: 20804623 PMC2994380

[87] Yatzidis H Dombros NV Digenis GE. On the usefulness of Glycylglycine in hemodialysis and peritoneal Dialysis solutions. ASAIO J. (1996) 42:984–92. doi: 10.1097/00002480-199642060-00011, PMID: 8959273

